# Effects of N-Acetylcysteine and N-Acetylcysteine Amide on Erythrocyte Deformability and Oxidative Stress in a Rat Model of Lower Extremity Ischemia-Reperfusion Injury

**DOI:** 10.1155/2020/6841835

**Published:** 2020-09-29

**Authors:** Gokhan Erol, Hakan Kartal, Faruk M. Comu, Erdem Cetin, Ertan Demirdas, Huseyin Sicim, Celal S. Unal, Celalettin Gunay, Bilgehan S. Oz, Cengiz Bolcal

**Affiliations:** ^1^Department of Cardiovascular Surgery, Gulhane Education and Research Hospital, Ankara, Turkey; ^2^Department of Physiology, Kırıkkale University Medical Faculty, Kırıkkale, Turkey; ^3^Department of Cardiovascular Surgery, Karabük Training and Research Hospital, Karabük, Turkey

## Abstract

N-acetylcysteine (NAC) is an antioxidant which works as a free radical scavenger and antiapoptotic agent. N-acetylcysteine-amide (NACA) is a modified form of NAC containing an amide group instead of a carboxyl group of NAC. Our study aims to investigate the effectiveness of these two substances on erythrocyte deformability and oxidative stress in muscle tissue. *Materials and Methods*. A total of 24 Wistar albino rats were used in our study. The animals were randomly divided into five groups as control (*n*: 6), ischemia (*n*: 6), NAC (*n*: 6), and NACA (*n*: 6). In the ischemia, NAC, and NACA groups, 120 min of ischemia and 120 min of reperfusion were achieved by placing nontraumatic vascular clamps across the abdominal aorta. The NAC and NACA groups were administered an injection 30 min before ischemia (100 mg/kg NAC; 100 mg/kg NACA; intravenous). Blood samples were taken from the animals at the end of the ischemic period. The lower extremity gastrocnemius muscle was isolated and stored at −80 degrees to assess the total antioxidant status (TAS), total oxidant status (TOS), and oxidative stress index (OSI) values and was analyzed. *Results*. The erythrocyte deformability index was found to be statistically significantly lower in rats treated with NAC and NACA before ischemia-reperfusion compared to the groups that received only ischemia-reperfusion. In addition, no statistically significant difference was found between the control group and the NAC and NACA groups. The groups receiving NAC and NACA before ischemia exhibited higher total antioxidative status and lower total oxidative status while the oxidative stress index was also lower. *Conclusion*. The results of our study demonstrated the protective effects of NAC and NACA on erythrocyte deformability and oxidative damage in skeletal muscle in lower extremity ischemia-reperfusion. NAC and NACA exhibited similar protective effects on oxidative damage and erythrocyte deformability.

## 1. Introduction

Cellular damage following reperfusion of ischemic tissues of the lower extremities is a common and critical clinical condition. Metabolites such as free oxygen radicals spread throughout the body through systemic circulation due to the reperfusion that occurs with restored blood flow. Unpaired electrons and free radicals are highly reactive and are easily involved in chemical reactions with almost all cellular components (lipids, proteins, complex carbohydrates, and nucleic acids) in the body. These reactions occur via a chain of oxidative reactions and cause tissue injury. The highly unsaturated fatty acids found in the cellular membranes are the most sensitive macromolecules to oxidative damage in cells. Endothelial cells, macrophages, neutrophils, and neuronal cells produce superoxide (0^2−^) and NO, and these radicals can combine to form peroxynitrite anion (ONOO-). Peroxynitrite induces lipid and protein peroxidation, causing direct cellular injury and damage to nucleic acids [1, 2].

Many trials have been performed on various substances that may have a protective effect on lower extremity ischemia-reperfusion injury [3]. There are also many studies showing that reperfusion injury following lower extremity ischemia causes erythrocyte deformation [4].

N-Acetylcysteine (NAC), a well-known thiol-containing antioxidant, has been widely used in clinics for more than 50 years [5, 6]. N-Acetylcysteine, a precursor of glutathione (GSH), is an FDA-approved drug [7]. NAC acts as a powerful scavenger of free radicals due to its nucleophilic reactions with reactive oxygen species [8]. However, due to the very low bioavailability of NAC, the modified compound N-acetylcysteine amide (NACA) has been developed and is more membrane permeable than NAC [9]. N-Acetylcysteine amide (NACA), also known as AD4, is a modified form of N-acetylcysteine containing an amide group instead of the carboxyl group of NAC. It was designed and synthesized with the possibility that neutralizing the carboxyl group would aid in its passage through cell membranes [10].

The main aim of this study was to investigate the effectiveness of N-acetylcysteine (NAC) and N-acetylcysteine amide (NACA) in erythrocyte deformability in lower extremity ischemia-reperfusion injury and to determine the relationship with oxidative stress in skeletal muscle tissue.

## 2. Materials and Methods

### 2.1. Animals and Experimental Protocol

All chemicals used in the study were supplied without the support of any institution or company (N-acetylcysteine amide and N-acetylcysteine, Sigma-Aldrich).

The study protocol was approved by the animal experiments committee of Gulhane Training and Research Hospital. Blood samples were analyzed in the physiology laboratory of Kırıkkale University. All procedures were carried out in accordance with the standards for the Care and Use of Laboratory Animals.

In the experiment protocol, 24 male Wistar albino rats weighing 250–350 g were used. The animals were kept in a special standard cage, each containing three rats, with 50% air humidity at 21–24°C for seven days. The 12-hour auto-light/dark cycle was maintained. All rats were able to reach standard food pellets and fresh drinking water throughout the study period. Later, the animals were randomized into four groups, each with six rats (one cage having three rats each).

### 2.2. Technical Procedure

General anesthesia was performed with Ketamine (90 mg/kg) + Xylazine (10 mg/kg) before surgery. All groups were administered 100 IU/kg heparin intravenous bolus 30 min before the surgical procedure. Next, the excess fur was removed by trimming the abdomen with a special electric animal shaver. Before the surgery, the surgical site was sterilized with an antiseptic solution. Anesthetic agents were tested for sufficiency before the procedure. The rats were subjected to a midline laparotomy. The infrarenal abdominal aorta was explored following the removal of the intestines with the aid of wet gauze. An atraumatic microvascular clamp was placed across the abdominal aorta (AA). After 120 min of ischemia, the microvascular clamp was removed from the AA, and reperfusion was provided for 120 min. Aortic ischemia was confirmed with the disappearance of pulsation in the distal aorta during the clamping process while aortic reperfusion was confirmed with pulsation in the distal aorta after declamping. Laparotomy and abdominal aortic dissection were performed equally (240 min) on rats in the control group, but no I/R was induced. During the period of I/R, saline was applied to the peritoneal cavity and the abdominal incision was closed temporarily by wrapping with wet gauze to minimize heat and fluid loss following clamping and declamping of the AA [11].

#### 2.2.1. Control Group (Gr Control, *n* = 6)

In this group, only midline abdominal laparotomy was performed and then closed without abdominal aortic ischemia. Two hours after the procedure, the rats were sacrificed under general anesthesia.

#### 2.2.2. Ischemia Group (Gr Ischemia, *n* = 6)

In this group, the rats were subjected to midline abdominal laparotomy and clamping of the abdominal aorta without the injection of N-acetylcysteine or N-acetylcysteine-amide. Following 120 min of ischemia and 120 min of reperfusion, the rats were sacrificed under anesthesia.

#### 2.2.3. NAC Group (Gr NAC, *n* = 6)

In this group, the rats were administered 100 mg/kg (0.2 ml) of N-acetylcysteine intravenously 30 min before ischemia. The rats were subjected to midline abdominal laparotomy and then a microvascular clamp was placed on the abdominal aorta. Following 120 min of ischemia and 120 min of reperfusion, the rats were sacrificed under anesthesia.

#### 2.2.4. NACA Group (Gr NACA, *n* = 6)

In this group, the rats received 0.2 ml of N-acetylcysteine amide (100 mg/kg) intravenously 30 min before ischemia. The rats were subjected to a midline abdominal laparotomy with a microvascular clamp placed across the abdominal aorta. After 120 min of ischemia and 120 min of reperfusion, the rats were sacrificed under anesthesia.

Blood samples were taken from the vena cava inferior from all subjects under anesthesia. Erythrocyte suspensions of 5% hematocrit with phosphate-buffered saline (PBS) were used for deformability measurements.

Tissues were taken from gastrocnemius muscle from all subjects under anesthesia. The hind limb tissue samples were enumerated and placed in a sterile Eppendorf tube and stored at −80°C until analysis of total antioxidant/oxidant status and oxidative stress index.

### 2.3. Deformability Measurements

A constant flow filtrometer system was used to measure erythrocyte deformability. Erythrocyte packs that were prepared and stored at room temperature (25 C^0^) were resuspended in PBS buffer to a 5% HTC solution. Nuclepore polycarbonate membrane filters, 25 mm in diameter, with a pore size of 5 *μ*m, were used. 1.5 ml/min filtration pressure cmH_2_O was measured by providing a flow rate. Actual pressure changes were monitored after they were transferred to the computer via the Biopac Data Acquisition System:

 
**PL**: erythrocyte suspension filtration pressure
 
**PT**: buffer solution filtration pressure
  relative pressure values were calculated as **Rrel**: P_Erythrocyte_/P_Buffer_

The relative pressure values obtained give us insight into the capacity of erythrocyte deformability. Such an increase indicates a negative effect on the capacity of erythrocyte deformability.

### 2.4. Homogenization of Tissues

The hind limb tissue was collected in a sterile Eppendorf tube and was stored at −80°C until total antioxidant/oxidant status and oxidative stress index analysis. Tissues were quickly weighed on a precise scale without allowing them to dissolve. An 80–100 mg specimen was separated for analysis using a scalpel (PLUSMED®). The frozen tissue samples were crushed with liquid nitrogen in a porcelain mortar. The pulverized tissue was transferred to the homogenization tube adding 140 mM KCl solution per gram of tissue, with dilution 1/10. The tube was kept in a glass beaker filled with ice for 2 min at 50 rpm (rpm) before and after homogenization with the homogenizer to prevent the temperature rise during homogenization. Homogenates were transferred to Eppendorf tubes. The tubes were covered with paraffin and then centrifuged at 3,000 rpm for 10 min. After centrifugation, the supernatant was collected in another Eppendorf tube and prepared for measuring total oxidative status (TOS) and total antioxidant status (TAS) [12].

### 2.5. TAS Measurement

Samples were analyzed on the fully automated Mindray BS300 device with the Relassay kit. 300 *μ*L of reagent 1 (measurement buffer) and 30 mcL of the sample were mixed, and absorbance was measured at 660 nm after 30 sec. Next, 45 *μ*L of reagent 2 (colored 2,2-azino-bis-3-ethylbenzothiazoline-6-sulfonic acid) (ABTS) was added to the mixture, and absorbance was measured at 660 nm after 5 min of incubation. For standard measurement, the Trolox Eq solution at a concentration of 1 mmol/L was equally used instead of the sample. The first and second measurements were performed three times, and their averages were calculated. The absorbance change (ΔAbs) was calculated by subtracting the first absorbance value (A1) from the second absorbance value (A2). TAS levels were calculated using the formula given in the kit and expressed as mmol Trolox Eq/L:

(1) $$\begin{array}{c} \text{TAS}=\frac{\Delta {\text{AbsH}}_{2}\text{O}-\text{Abs sample}}{\Delta {\text{AbsH}}_{2}\text{O}-\text{Abs standard}}. \end{array}$$

### 2.6. TOS Measurement

Samples were analyzed on the fully automated Mindray BS300 device with the Relassay kit. 300 *μ*L of reagent 1 (measurement buffer) and 45 *μ*L of the sample were mixed, and the first reading was performed at 530 nm after 30 sec. Next, 15 uL of reagent 2 (prochromogenic solution) was added to the mixture, and the second reading was performed at 530 nm after 5 min of incubation. A standard solution containing 10 *μ*mol/L hydrogen peroxide (H_2_O_2_) (equivalent/liter) supplied in the kit was used for standard measurement. The first and second measurements were performed three times, and their averages were calculated. The absorbance change (ΔAbs) was calculated by subtracting the first absorbance value (A1) from the second absorbance value (A2). The TOS levels were calculated using the formula provided in the kit and expressed as mmol H_2_O_2_ Eq/L:

(2) $$\begin{array}{c} \text{TOS}=\frac{\Delta \text{Abs sample}}{\Delta \text{Abs standard}}\times \text{standard concentration }\frac{10\;\mu \text{mol}}{\text{L}}. \end{array}$$

### 2.7. Oxidative Stress Index (OSI)

The ratio of TOS to TAS was accepted as the oxidative stress index (OSI). For calculation, the resulting TAS unit was converted to *μ*mol/L and the OSI value was calculated according to [13–15]

(3) $$\begin{array}{c} \text{OSI}\left(\text{arbitrary unit}\right)=\frac{\text{TOS}\left(\mu \text{mol }{\text{H}}_{2}{\text{O}}_{2}\;\text{equivalent}/\text{L}\right)}{\text{TAC}\left(\mu \text{mol Trolox }\text{equivalent}/\text{L}\right)}. \end{array}$$

### 2.8. Statistical Analysis

Descriptive statistics of continuous variables were expressed as mean and standard deviation. Kolmogorov–Smirnov test was used to compare the distribution of continuous variables. Levene's test was used to test variance homogeneity. One-way ANOVA was used to evaluate the difference between the mean values of erythrocyte deformability in the control, ischemia, NAC, and NACA groups. Bonferroni adjustment and planned contrast tests were used for multiple comparisons. Clinical significance was evaluated using omega squared (*ω*^2^) to measure the overall effect of the study (for ANOVA) (limit values: 0.01 small, 0.06 medium, and 0.14 large) [16]. The Pearson correlation coefficient (*r*) was used as the effect size for paired comparisons obtained by planned contrast to determine the clinical effectiveness of the NAC and NACA treatments. Pearson correlation coefficient (*r*) (0.10 small, 0.30 medium, and 0.50 large) was evaluated according to the limit values recommended by Cohen (1988) [17].

Mean, standard deviation, median, interquartile difference (interquartile range: IQR), and 95% confidence interval were used as descriptive statistics for continuous data in the evaluation of TAS, TOS, and OSI. Kolmogorov–Smirnov test was used to test the normal distribution of the data. The means of the oxidative stress index (OSI) showing normal distribution in the groups were compared with one-way ANOVA while Bonferroni correction was used for multiple comparisons if a significant difference was achieved. The Kruskal–Wallis test was used to compare the distribution of total antioxidant status (TAS) and total oxidant status (TOS) values that did not meet the parametric assumptions in the groups. In the case of statistical significance, the Mann–Whitney *U*-test with Bonferroni correction was used to assess the source of the difference (type-I error 0.05/6 = 0.008) [15]. Clinical significance was determined using partial-eta-square (partial-*η*^2^) for one-way ANOVA and eta-square (*η*^2^) for Kruskal–Wallis according to the limit values (0.0099 small, 0.0588 medium, and 0.1379 large) recommended by Cohen (1988) [16–18].

All statistical analyses were performed using the IBM SPSS Statistics version 25 software package (IBM SPSS Inc. Chicago. IL). The significance level was accepted as 0.05.

## 3. Results

**Figure 1:**
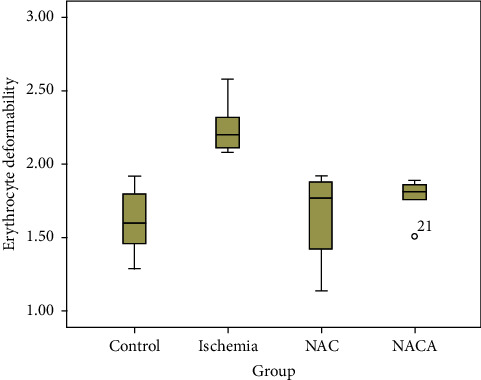
Box plot graph showing erythrocyte deformability distributions in groups.

**Figure 2:**
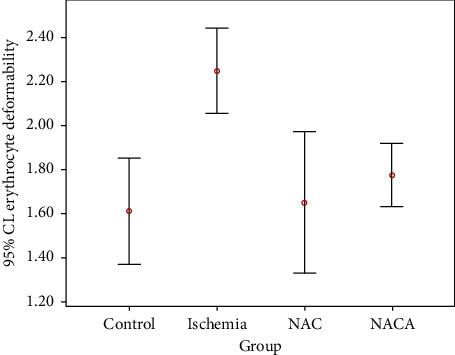
Error bar graph showing erythrocyte deformability distributions in groups with 95% confidence interval.

In Table 1, the mean values of erythrocyte deformability were compared were with one-way ANOVA in all four groups. Levene's test demonstrated that the group variances were homogeneous. The one-way ANOVA test revealed a significant difference in the mean values of erythrocyte deformability between the groups (F (3.20) = 10.381, *p* < 0.001, *ω*^2^ = 0.54). Bonferroni correction was used for multiple comparisons to determine the source of the difference. The paired comparisons revealed that the mean values in the ischemia group (2.247 ± 0.18) were statistically significantly higher than in the control (1.61 ± 0.23), NAC (1.65 ± 0.31), and NACA (1.77 ± 1.14) groups (1-2 (*p* < 0.001), 2-3 (*p*=0.001), and 2–4 (*p*=0.009), respectively). There was no significant difference between the control group (1.61 ± 0.23) and NAC (1.65 ± 0.31) and NACA (1.77 ± 1.14) groups (*p*=1.000). There was also no significant difference between the NAC (1.65 ± 0.31) and NACA (1.77 ± 1.14) groups (*p*=1.000) (Table 1, Figures 1 and 2).

Clinical significance was evaluated using the omega squared (*ω*^2^) effect size index for ANOVA. The effect size of our study was high (*ω*^2^ = 0.54 > 0.14). Pearson correlation coefficient (*r* = 0.72), used as an impact size index, was calculated to measure the effectiveness of NAC treatment. It was found higher than the limit values recommended by Cohen's (1988) (*R* = 0.72 > 0.50). Pearson correlation coefficient (*r* = 0.64), used as an impact size index, was calculated to measure the effectiveness of NACA treatment. It was found higher than the limit values recommended by Cohen's (1988) (*r* = 0.64 > 0.50).

**Figure 3:**
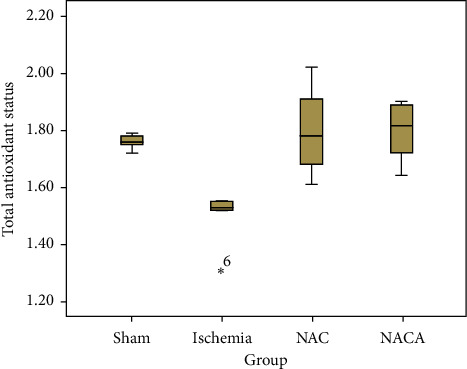
Box plot chart of TAS.

Table 2 shows the analysis results obtained by comparing the distributions of total antioxidant status (TAS) and total oxidant status (TOS) values in the sham, ischemia, NAC, and NACA groups using the Kruskal–Wallis test. A statistically significant difference was observed when the mean TAS values in the sham, ischemia, NAC, and NACA groups were compared (*χ*2 = 12.956; *p*=0.005). The Mann–Whitney U multiple comparison test with Bonferroni correction was performed to find the source of the difference revealing that the mean of sham (1.760 ± 0.027) group was significantly higher than the mean of ischemia group (1.498 ± 0.093) (*p*=0.006) while the mean of ischemia (1.498 ± 0.093) group was significantly lower than the mean of NAC (1.802 ± 0.166) and NACA (1.800 ± 0.101) groups (*p*=0.006 and *p*=0.004, respectively). In addition, there was no significant difference between the mean of NAC and NACA groups (*p*=1.000) and other paired comparisons (*p* > 0.05) (Table 2, Figure 3).

Clinical significance was evaluated using eta-square (*η*^2^) for Kruskal–Wallis. The effect size, *η*^2^ = 0.553 > 0.1379, was found to be large according to Cohen's recommended limit values (0.0099 small, 0.0588 medium, and 0.11379 large).

**Figure 4:**
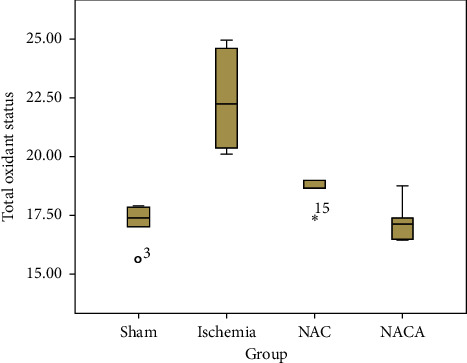
Box plot chart of TOS.

The mean values of total oxidant status (TOS) were compared, revealing a statistically significant difference between the four groups (*χ*2 = 15.756; *p*=0.001). The Mann–Whitney U multiple comparison test with Bonferroni correction was performed to find the source of the difference revealing that the mean of ischemia (22.425 ± 2.055) group was significantly higher than the means of sham (17.140 ± 0.898), NACA (17.225 ± 0.842), and NAC (18.532 ± 0.655) groups (*p*=0.006, *p*=0.006, and *p* = 0.004, respectively). There was no significant difference between the means of the NAC and NACA groups (*p*=0.028) and other paired comparisons (*p* > 0.05) (Table 2, Figure 4).

The effect size (*η*^2^ = 0.708 > 0.1379) was found to be large according to Cohen's recommended limit values (0.0099 small, 0.0588 medium, and 0.1379 large).

**Figure 5:**
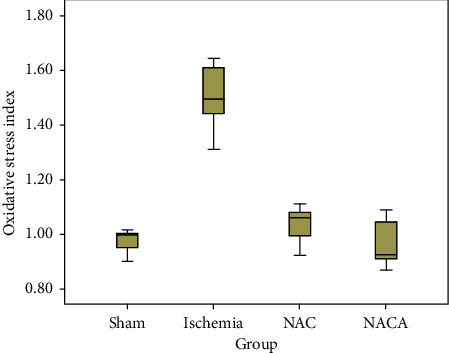
Box plot chart of OSI.

The mean oxidative stress index (OSI) values were compared using the one-way ANOVA test in all four groups. A statistically significant difference was found when the mean values of TAS in the sham, ischemia, NAC, and NACA groups were compared (F (3.18) = 48.112; *p* < 0.001). The Bonferroni correction, a multiple comparison test, was performed to find the source of the difference revealing that the mean of ischemia (22.425 ± 2.055) group was significantly higher than the means of sham (0.973 ± 0.047), NAC (1.033 ± 0.075), and NACA (0.960 ± 0.086) groups (*p* < 0.001). There was no significant difference between the means of the NAC and NACA groups (*p*=1.000) and other paired comparisons (*p*=1.000) (Table 2, Figure 5).

Clinical significance was evaluated using partial-eta-squared with ANOVA. The effect size, partial-*η*^2^ = 0.889 > 0.1379, was found to be large according to Cohen's recommended limit values (0.0099 small, 0.0588 medium, and 0.11379 large).

## 4. Discussion

In the present study, we aimed to compare the effect of NAC and NACA on erythrocyte deformability and tissue oxidative stress levels in lower extremity IR injuries in a rat model. Our study results showed that NAC and NACA exerted a protective effect on erythrocyte deformation and tissue oxidative stress levels in lower extremity IR injuries in rats.

Erythrocytes must have the capacity to move through the end organ capillaries to deliver oxygen and vital molecules to the tissues and clear metabolic waste through the final organ capillaries. This ability is called “deformability” and gains more importance in microcirculation. Modified erythrocyte deformability is critical not only for the oxygen delivery capacity of erythrocytes but also for the survival of circulating erythrocytes [19, 20]. The deformability of erythrocytes is the key factor for the regulation of the normal microvascular circulation. The viscoelastic properties of the erythrocyte membrane are the main determinant of the deformability of red blood cells [21].

I/R primarily causes microcirculation damage. Leukocyte–endothelial interactions trigger the release of reactive oxygen species and elastase causing transendothelial migration and tissue damage [22]. The main features of reperfusion injury include cell swelling degeneration of the cytoskeleton structure and loss of selective permeability in the cell membrane, all of which result in reduced capillary blood flow and tissue edema [23].

In our study, there was a statistically significant difference between the control group and the ischemia group with a higher deformability index in the ischemia group. Thus, the negative effect of lower extremity ischemia-reperfusion injury on erythrocyte deformability was demonstrated.

Erythrocytes have a biconcave disc shape with a diameter of about 8 microns. On the other hand, the smallest capillary diameter in microcirculation is only 3 microns. Beyond these capillaries the diameter of the thick endothelial cell spaces in the spleen is as small as 0.5 *μ*m [24]. Reduced erythrocyte deformability may result in increased blood viscosity obstruction in microvessels impaired perfusion and ischemia. Although the underlying mechanisms are partially explained, ischemia-reperfusion affects blood and plasma viscosity, which is important for erythrocyte deformability and aggregation [25, 26].

A study conducted by Pace et al. found that patients with sickle cell disease receiving NAC treatment had decreased formation of dense and irreversible sickle cells normalized glutathione levels and fewer vaso-occlusive attacks at doses of 2400 mg/day [27]. A study by Amen et al. investigated the potential use of NAC as an additive in the preservation solution to improve RBC performance after transfusion and reported that it could be used as an additive in the preservation solution to preserve RBC [28]. A study conducted by Ortaloni et al. on 30 patients with septic shock found that lipoperoxidative indexes and erythrocyte membrane stiffness were significantly decreased by the NAC treatment when compared with the control group [29]. In our study, we found that NAC, which differently exhibits protective effects on erythrocytes compared to the studies of Pace et al. [27], Amen et al. [28] and Ortolani et al. [29], has a protective effect on erythrocyte deformability in lower extremity ischemia-reperfusion injury.

A study by Goyal et al. showed that NACA attenuated oxidative stress and apoptosis in doxorubicin + trastuzumab-mediated cardiac dysfunction in a murine model, alleviating the cardiotoxic side effects associated with chemotherapy [30]. The results of our study demonstrate that NACA which was reported to be antiapoptotic and antioxidative by Goyal et al. exhibits a protective effect on erythrocyte deformability in the lower extremity ischemia-reperfusion.

A study by Gunther et. al. showed that NACA exhibited protective properties against brain neuronal degeneration and apoptosis in rats suffering focal brain trauma. The magnitude of the effect was attributed to the fact that NACA has a higher membrane and blood-brain barrier permeability than NAC which has limited but well-documented neuroprotective effects after the experimental central nervous system ischemia while having a low bioavailability [31]. On the other hand, our results did not reveal a significant difference in the erythrocyte deformability index although the study conducted by Gunther et al. found that NACA had higher tissue perfusion compared to NAC. Despite no statistical difference, NAC was found even closer to the control group than to NACA along with a higher Pearson correlation coefficient.

Methods that measure the total content of oxidant and antioxidant molecules rather than individually have recently gained more popularity for evaluating oxidative stress and antioxidant capacity in the body [32, 33].

For this reason, the values of total oxidant status, total antioxidant status, and oxidative stress were analyzed to evaluate the oxidative changes and changes in antioxidants caused by reperfusion injury in skeletal muscle. In addition, we think that the oxidative status occurring in the tissue is more significant since our study mainly focuses on microvascular circulation.

In our study, NAC and NACA were observed to improve total oxidative status in the reperfused skeletal muscle with similar results found in the sham group. The oxidative stress index (OSI), which is the ratio of total oxidative status and total antioxidative status in the tissue, is a better indicator for the level of tissue damage associated with reperfusion compared to TAS and TOS as it reflects the balance between the oxidants and antioxidants in the tissue. For example, tissue damage is inevitable when a bioactive agent increases the total antioxidative capacity in the tissue but results in a higher increase in the total oxidative status. In our study, NAC and NACA significantly reduced the oxidative stress index, which increased due to reperfusion injury in the ischemia group. This result shows that NAC and NACA protect the tissue against oxidative damage and also provide a balance between antioxidants and oxidants. Our study demonstrated that NAC and NACA reduced the tissue oxidative stress levels and erythrocyte deformability in skeletal muscle reperfusion injury. There was no significant difference between NAC and NACA in terms of effectiveness although results of NAC are better. Dose studies and clinical trials are needed to investigate the effectiveness of NAC and NACA on erythrocyte deformability and oxidative activity.

## 5. Limitations

Our study had some limitations. The first limitation was the small sample size followed by the absence of a dosing study due to the small sample size.

## 6. Conclusion

In conclusion, the present study demonstrated that NAC and NACA caused a statistically significant decrease in deformability index and oxidative stress compared to the group with induced ischemia-reperfusion on erythrocytes in the event of lower extremity ischemia-reperfusion. Although the deformability index and oxidative stress index were better in NAC-treated rats than NACA-treated rats, no statistically significant difference was found. NAC and NACA may be used as antioxidant drugs in the near future.

## Figures and Tables

**Table 1:** Comparison of erythrocyte deformability values between the groups.

	Control (1) mean ± SD (*n* = 6)	Ischemia (2) mean ± SD (*n* = 6)	NAC (3) mean ± SD (*n* = 6)	NACA (4) mean ± SD (*n* = 6)	Test statistics	Source of difference (pairwise comparisons)
Erythrocyte deformability	1.61 ± 0.23	2.247 ± 0.18	1.65 ± 0.31	1.77 ± 1.14	F(3.20) = 10.381^*∗*^*p*=0.001^*∗∗*^ *ω*^2^ = 0.54^*∗∗∗*^	1-2 (*p* < 0.001) 1–3 (*p*=1.000) 1–4 (*p*=1.000) 2-3 (*p*=0.001) (*r* = 0.72)^*∗∗∗∗*^ 2–4 (*p*=0.009) (*r* = 0.64)^*∗∗∗∗*^ 3-4 (*p*=1.000)

^*∗*^One-way ANOVA statistics values 1–4. ^*∗∗*^The mean significant difference at the level of 0.05. (*p* < 0.05). ^*∗∗∗*^Omega squared effect size index. ^*∗∗∗∗*^Pearson correlation coefficient effect size index.

**Table 2:** Distribution of TAS, TOS, and OSI variables in groups.

	Sham (1) (mean ± Sd) (median ± IQR) *n* = 5	Ischemia (2) (mean ± Sd) (median ± IQR) *n* = 6	NAC (3) (mean ± Sd) (median ± IQR) *n* = 5	NACA (4) (mean ± Sd) (median ± IQR) *n* = 6	Test statistics values	Source of the difference
TAS	1.760 ± 0.027 (1.726–1.794)	1.498 ± 0.093 (1.414–1.742)	1.802 ± 0.166 (1.403–2.081)	1.800 ± 0.101 (1.640–1.925)	*χ*2(3) = 12.956^*∗*^ *p*=0.005^*∗∗∗*^ *η*2 = 0.553^*∗∗∗∗∗∗*^	1 > 2 (*p*=0.006)^*∗∗∗∗*^ 1 < 3 (*p*=0.675) 1 < 4 (*p*=0.314) 2 > 3 (*p*=0.006)^*∗∗∗∗*^ 2 > 4 (*p* < 0.004)^*∗∗∗∗*^ 3 > 4 (*p*=1.000)

TOS	17.140 ± 0.984 (15.584–19.495)	22.425 ± 2.055 (20.267–24.582)	18.532 ± 0.652 (17.704–19.759)	17.225 ± 0.842 (16.341–18.108)	*χ*2(3) = 15.756 *p*=0.001^*∗∗∗*^ *η*2 = 0.708^*∗∗∗∗∗∗*^	1 < 2 (*p*=0.006)^*∗∗∗∗*^ 1 < 3 (*p*=0.047) 1 < 4 (*p*=0.715) 2 > 3 (*p*=0.006)^*∗∗∗∗*^ 2 > 4 (*p* < 0.004)^*∗∗∗∗*^ 3 > 4 (*p*=0.028)

OSI	0.973 ± 0.047 (0.896–1.095)	1.498 ± 0.122 (1.289–1.564)	1.033 ± 0.075 (0.916–1.269)	0.960 ± 0.086 (0.863–1.079)	F(3.18) = 48.112∗∗ *p* < 0.001^*∗∗∗*^ *η*2 = 0.889^*∗∗∗∗∗∗∗*^	1 < 2 (*p* < 0.001)^*∗∗∗∗∗*^ 1 < 3 (*p*=1.000) 1 > 4 (*p*=1.000) 2 > 3 (*p* < 0.001)^*∗∗∗∗∗*^ 2 > 4 (*p* < 0.001)^*∗∗∗∗∗*^ 3 > 4 (*p*=1.000)

^*∗*^F test statistics value. ^*∗∗*^Kruskal–Wallis test statistics value. ^*∗∗∗*^Statistical significance at the level of 0.05 (*p* < 0.05). ^*∗∗∗∗*^Bonferroni correction used as a post hoc test. ^*∗∗∗∗∗*^The Bonferroni-corrected Mann–Whitney U-test used as a post hoc test. ^*∗∗∗∗∗∗*^Eta-square (partial-*η*^2^) effect size index for Kruskal–Wallis. ^*∗∗∗∗∗∗∗*^Partial-eta-square (partial-*η*2) effect size index for ANOVA.

## Data Availability

No data were used to support this study.
